# Clusterin/Akt Up-Regulation Is Critical for GATA-4 Mediated Cytoprotection of Mesenchymal Stem Cells against Ischemia Injury

**DOI:** 10.1371/journal.pone.0151542

**Published:** 2016-03-10

**Authors:** Bin Yu, Yueting Yang, Huan Liu, Min Gong, Ronald W. Millard, Yi-Gang Wang, Muhammad Ashraf, Meifeng Xu

**Affiliations:** 1 Department of Pathology and Laboratory Medicine, University of Cincinnati Medical Center, Cincinnati, Ohio, United States of America; 2 Department of Pharmacology & Cell Biophysics, University of Cincinnati Medical Center, Cincinnati, Ohio, United States of America; Georgia Regents University, UNITED STATES

## Abstract

**Background:**

Clusterin (Clu) is a stress-responding protein with multiple biological functions. Our preliminary microarray studies show that clusterin was prominently upregulated in mesenchymal stem cells (MSCs) overexpressing GATA-4 (MSC^GATA-4^). We hypothesized that the upregulation of clusterin is involved in overexpression of GATA-4 mediated cytoprotection.

**Methods:**

MSCs harvested from bone marrow of rats were transduced with GATA-4. The expression of clusterin in MSCs was further confirmed by real-time PCR and western blotting. Simulation of ischemia was achieved by exposure of MSCs to a hypoxic environment. Lactate dehydrogenase (LDH) released from MSCs was served as a biomarker of cell injury and MTs uptake was used to estimate cell viability. Mitochondrial function was evaluated by measuring mitochondrial membrane potential (ΔΨm) and caspase 3/7 activity.

**Results:**

(1) Clusterin expression was up-regulated in MSC^GATA-4^ compared to control MSCs transfected with empty-vector (MSC^Null^). MSC^GATA-4^ were tolerant to 72 h hypoxia exposure as shown by reduced LDH release and higher MTs uptake. This protection was abrogated by transfecting Clu-siRNA into MSC^GATA-4^. (2) Exogenous clusterin significantly decreased LDH release and increased MSC survival in hypoxic environment. Moreover, ΔΨm was maintained and caspase 3/7 activity was reduced by clusterin in a concentration-dependent manner. (3) p-Akt expression in MSCs was upregulated following pre-treatment with clusterin, with no change in total Akt. Moreover, cytoprotection mediated by clusterin was partially abrogated by Akt inhibitor LY294002.

**Conclusions:**

Clusterin/Akt signaling pathway is involved in GATA-4 mediated cytoprotection against hypoxia stress. It is suggested that clusterin may be therapeutically exploited in MSC based therapy for cardiovascular diseases.

## Introduction

Bone marrow stem cell (BMSC) based therapeutics is an emerging therapy with potential to salvage cardiomyocytes during acute myocardial infarction (AMI). Cell therapy promotes regeneration and endogenous repair of damaged myocardium in patients. The safety and feasibility of administration of bone marrow derived mesenchymal stem cells (MSCs) in patients has been investigated. Intracoronary infusion [1] or intramyocardial injection [2] of autologous bone marrow derived MSC in patients shortly after AMI is feasible and no significant adverse events related to MSC treatment were observed. However, the benefits of cell-based therapies for adjunctive treatment of AMI in multiple clinical trials still remain controversial. One meta-analysis showed that BMSCs attenuated infarct size expansion and contributed to myocardial regeneration, resulting in overall improvement of heart function [3]. However, another meta-analysis showed that intracoronary infusion of bone marrow-derived mononuclear cell (BMMNC) in patients with AMI did not enhance cardiac function on MRI-derived parameters, nor did it improve clinical outcome [1].

It has been shown that a better clinical outcome is associated with the number and characteristics of transplanted cells. An intracoronary stem cell therapy in patients with AMI showed that the improved left ventricle ejection fraction (LVEF) was dependent on the number of transplanted cells [4]. The LVEF was significantly improved in the patients treated with a high cell number of autologous BMSCs [4]. More importantly, the results from a CCTRN TIME (Cardiovascular Cell Therapy Research Network Timing in Myocardial Infarction Evaluation) trial suggested that the characteristics of BMSCs were closely associated with the clinical outcome in patients with ST segment-elevation-myocardial infarction [5]. The low retention of cells in the delivery sites has been identified as the most critical problem for advancement of cardiac regenerative medicine [6]. Several experiments have demonstrated that strategies combining cell therapy with gene therapy might improve stem cell tolerance to ischemic environment [7, 8].

We have successfully engineered bone marrow derived MSCs overexpressing GATA-4 (MSC^GATA-4^) which survived far better in ischemic myocardium than control MSCs transduced with an empty-vector (MSC^Null^) [9]. The cytoprotection was associated with upregulation of anti-apoptotic proteins in MSC^GATA-4^ regulated by expression of multiple miRs [10]. The microarray data also indicated that clusterin (Clu) was up-regulated in MSC^GATA-4^. Clusterin is a multifunctional glycoprotein which is widely distributed in many tissues and regulated by a variety of environmental changes. Clusterin can bind to aggregated LDL in human plasma and plays a protective role against LDL aggregation [11]. Clusterin protects cells against both apoptosis or/and necrosis induced by genotoxic stress, heat stress, oxidative stress, or growth factor withdrawal [12–15]. Clusterin inhibited ischemia-induced death in H9c2 cells and in isolated adult ventricular rat cardiomyocytes [16]. Moreover, significant changes in clusterin level have been detected in patients with AMI [17]. Administration of human clusterin significantly reduced infarct size and death of animals in experimental MI [18]. It has also been shown that clusterin has a protective effect on cardiomyocytes after AMI in rat. Further studies imply that the anti-apoptotic effect of clusterin on oxidative stress-induced apoptosis of cardiomyocytes is mediated at least in part through Akt/GSK-3β signaling [19]. In this study, we are proposing that GATA-4 overexpression mediated cytoprotection of hypoxic MSCs is triggered by clusterin/Akt signaling pathway.

## Methods

### Ethics statement

All animals were treated following the guidelines for the Care and Use of Laboratory Animals prepared by the National Academy of Sciences and published by the National Institutes of Health (NIH publication No. 85–23, Revised 1996). The animal experimental protocol (Xu-05-10-19-01) was approved by the Institutional Animal Care and Use Committee of the University of Cincinnati.

### MSCs culture and transduction with GATA-4 plasmid as well as Clu-siRNA

MSCs obtained from femurs and tibias of Sprague-Dawley (SD) rats were cultured with Iscove's Modified Dulbecco's Medium (IMDM) supplemented with 10% FBS and penicillin (100 U/ml) and streptomycin (100 μg/ml). MSCs were transduced with GATA-4 using the murine stem cell virus (pMSCV) retroviral expression system (Clontech) [9]. Briefly, GP2-293 cells (Clontech) were first co-transfected with pMSCV-GATA-4-IRES-EGFP and pVSVG for 48 h. Supernatants collected from GP2-293 were filtered and added into MSCs culture (second passage) in the presence of 10 μg/ml polybrene (Sigma) for 12 h. Stable GATA-4-expressing clones were selected with puromycin (3 μg/ml) (Sigma) for 5 days. Positively transduced MSCs were confirmed using GATA-4 immunostaining. Control MSCs (MSC^Null^) were transduced with the empty vector (pMSCV-IRES-EGFP) and pVSVG.

Rat Clu-siRNA (Ambion, siRNA ID: s128555) was further transfected into MSC^GATA-4^ using Lipofectamine RNAiMAX (Invitrogen). Briefly, 3 × 10^4^ MSCs/well (6-well plate) were seeded in 2 ml of IMDM containing 10% fetal bovine serum (FBS) without any antibiotics. The cells were transfected with Clu-siRNA (75 pmol) when the cell density reached 50–60% confluence. The negative control cells were transfected with scramble-siRNA (MSC^NC-siRNA^). Transfected cells were harvested 48 h later.

### Cell ischemic model and the evaluation of cell injury

To simulate ischemic injury, the culture medium of MSCs was replaced with low glucose (1 g/L) DMEM and cells were placed in a hypoxia incubator (Sanyo, CO_2_/O_2_ incubator, MCO-18M) with 5% CO_2_, 94% N_2_ and 1% O_2_.

1. The release of lactate dehydrogenase (LDH) from MSCs was used to evaluate cell injury by using a commercially available kit according to the manufacturer’s instructions (Biovision).
2. Cell survival was estimated using a tetrazolium compound (MTs) in the CellTiter 96^®^ AQ_ueous_ One Solution cell proliferation assay kit (Promega).
3. ΔΨm of MSC was monitored by incubating with JC-1 (5 μmol) at 37°C for 15 min and images were taken using a invert fluorescent microscope (Olympus IX-71). JC-1 monomer (green) fluorescence was measured at λex = 488 nm / λem = 505 to 530 nm, and JC-1 J-aggregate (red) fluorescence was at λex = 543 nm / λem = 560 nm, respectively. The ratio of red to green fluorescence was determined and interpreted as ΔΨm. A decrease in the ratio indicated a loss of ΔΨm [20].
4. The activity of caspases 3 and 7 was assessed with the Caspase-Glo^®^ 3/7 Assay Kit (Promega).

The intensities of both fluorescence ranges of JC-1 and the luminescence were determined using a microplate M3 spectrophotometer (Molecular Devices, Sunnyvale, CA).

### Quantitation of mRNA expression using real-time PCR

Total RNA of cultured MSCs was extracted using Trizol reagent (Invitrogen), followed by DNAse treatment and purification using RNeasy mini column kit (Qiagen). An aliquot of the cDNA synthesized using the SuperScript^™^ III RNase H^−^ Reverse Transcriptase (Invitrogen) was amplified using *Taq* DNA polymerase (2.5 U) (Invitrogen). The primers of clusterin were provided by IDT, Inc. The specific sequences are: Clu-forward: ACA GTG TGC AAG GAG ATC CG; and Clu-reward TAG CCT GGG CAG GAT TGT TG. Quantitative PCR was performed on the iQ5 real-time system (Bio-Rad). PCR efficiency was evaluated against a standard curve of five serial dilution points. Data were analyzed using BioRad IQ software and mRNA was normalized to the housekeeping gene, GAPDH.

### Electroimmunoblotting

The proteins were isolated from MSCs and concentrations were quantified with Bio-Rad DC-Protein Assay Reagent (Bio-Rad). Denatured protein (25 μg) was electrophoresed using 12% sodium dodecyl sulfate—polyacrylamide gel electrophoresis (SDS—PAGE) and transferred to a polyvinylidene difluoride (PVDF) membrane. The membrane was blocked with 5% non-fat milk in Tris-buffer saline solution (pH 7.6) containing 0.05% Tween-20 (TBS/T), and then incubated in antibodies against clusterin, total Akt, p-Akt (1:1000), and β-actin (1:2000) (Cell Signaling) overnight at 4°C, respectively. The PVDF membrane was incubated with secondary antibody which conjugated with HRP at room temperature for 1 h, washed and developed with the ECL Prime kit (GE Healthcare). Densitometric analysis for the blots was performed with a computer program (Image Quant Solution).

### Statistical analysis

The parameters of cell injury, real-time PCR and western blot data, and all other quantitative data are presented as the mean ± SEM for measurements obtained from at least three independent replicates. When the *p* value was less than 0.05, the differences were considered significant. One-way analysis of variance and post-hoc least significant difference correction was used to compare the differences among groups.

## Results

### Hypoxia induced MSCs injury

**Fig 1:**
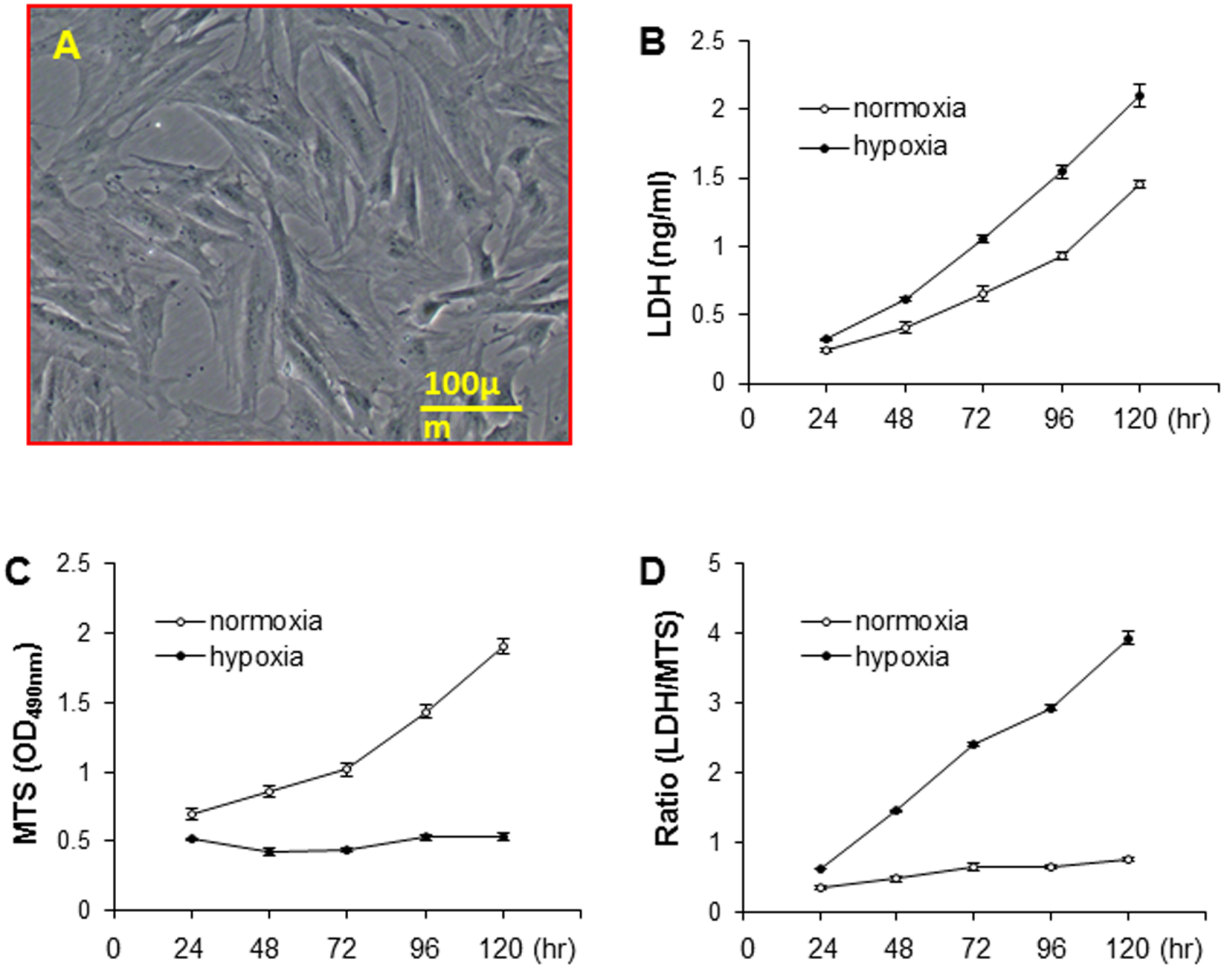
MSC injury following exposure to hypoxia from 24 h to 120 h. A. Morphology of MSCs in normoxia culture; B. LDH release; C. MTs intake; D. Ratio of LDH to MTs.

Bone marrow derived MSCs showed the plastic adherent properties and had a fibroblast-like morphology. The cell body had a large, round nucleus with a prominent nucleolus, which is surrounded by finely dispersed chromatin particles (Fig 1A). LDH release was significantly increased in MSCs subjected to the hypoxia environment in a time-dependent manner from 24 h to 120 h. However, LDH released from normal cultured MSCs was also increased (Fig 1B). To determine whether LDH release is related to cell number increase or cell injury, the number of surviving MSCs was calculated using MTs test. MTs uptake was increased in cells cultured in normoxia from 24 h to 120 h, indicating that MSCs were proliferating during these periods. However, MTs uptake was not significantly changed when MSCs were exposed to the hypoxia environment, indicating that the numbers of MSCs were not increasing, a consequence of cell injury due to hypoxia (Fig 1C). Therefore, the ratio of LDH to MTs intake was increased following exposure to hypoxia in a time dependent manner (Fig 1D).

### Overexpression of GATA-4 upregulated clusterin expression and increased MSC survival

**Fig 2:**
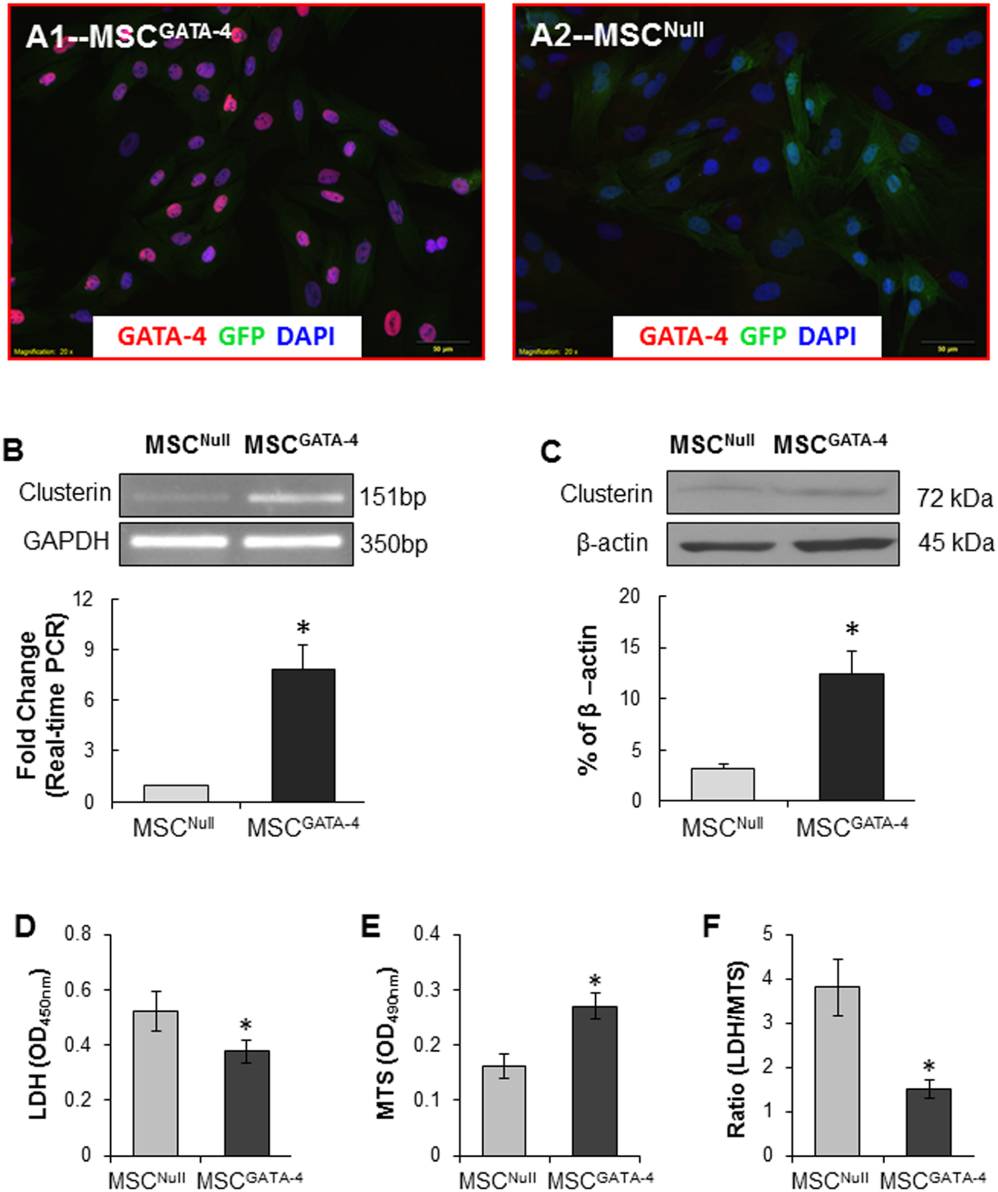
Characterization of MSC^GATA-4^. A. MSCs were immunostained with GATA-4. B. mRNA of clusterin in MSCs measured by conventional PCR and real-time PCR. C. Western blot of clusterin and its semi-quantitative data. D-F. The resistance of MSC^GATA-4^ against exposure to hypoxia for 72 h. *, *p* < 0.05 *vs* MSC^Null^.

No obvious differences were observed in morphology of MSCs transduced with GATA-4 compared to MSC^Null^ except the expression of GATA-4 in MSC^GATA-4^ (Fig 2A). Clusterin expression was significantly upregulated in MSC^GATA-4^ compared to MSC^Null^ both in mRNA (Fig 2B) and protein levels (Fig 2C). LDH release was significantly reduced and MTs uptake was significantly higher in MSC^GATA-4^ compared MSC^Null^ following exposure to hypoxia for 72 h (Fig 2D–2F).

**Fig 3:**
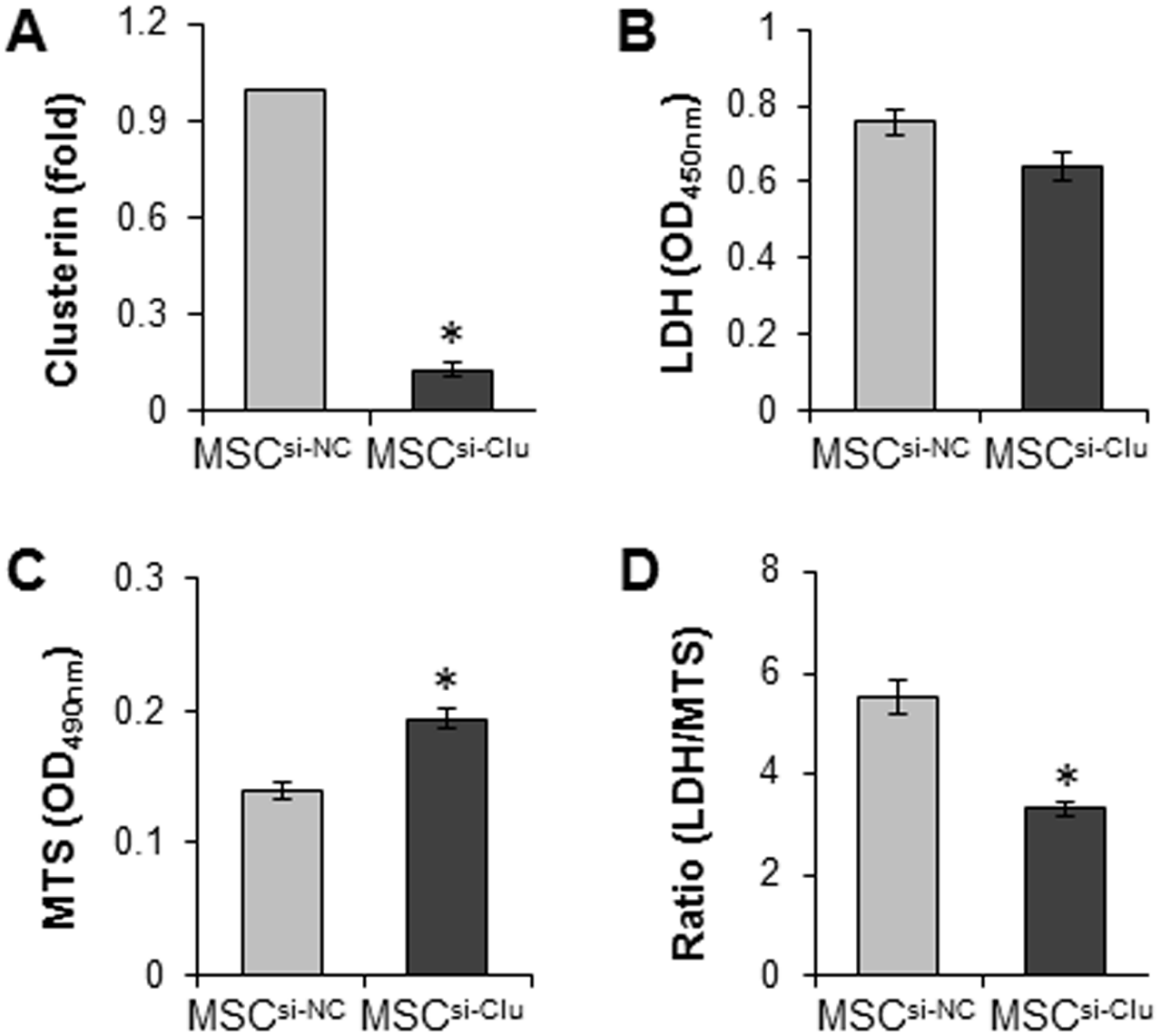
MSC^GATA-4^ transfected with Clu-siRNA (MSC^siRNA-Clu^) and transfected with negative control siRNA (MSC^siRNA-NC^) were exposed to hypoxia for 72 h. A. The expression of clusterin in MSC^siRNA-Clu^ and MSC^siRNA-NC^; B. LDH release; C. MTs intake; D. Ratio of LDH to MTs. *, *p* < 0.05 *vs* MSC^siRNA-NC^. MSC^si-Clu^ = MSC^siRNA-Clu^; MSC^si-NC^ = MSC^siRNA-NC^.

To test whether clusterin was involved in GATA-4 mediated cytoprotection, Clu-siRNA was introduced into MSC^GATA-4^ (MSC^siRNA-Clu^). After exposure to hypoxia, the expression of clusterin in MSC^siRNA-Clu^ was significantly reduced compared to the scramble-siRNA transfected cells (MSC^siRNA-NC^) (Fig 3A). Clu-siRNA partially abrogated cytoprotective responses in MSC^GATA-4^, including LDH release and MTs uptake (Fig 3B).

**Fig 4:**
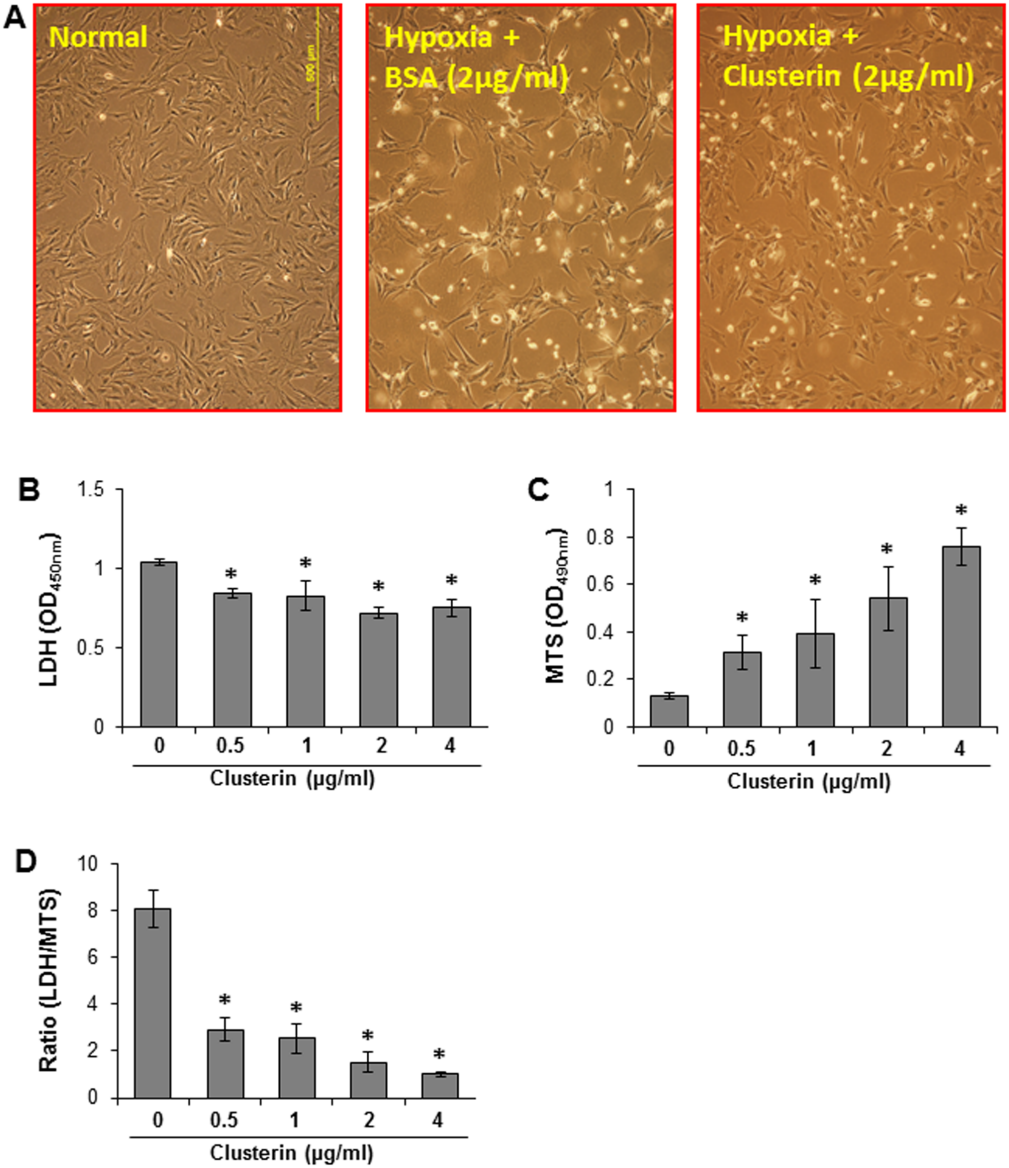
The protective effect of clusterin (0 ~ 4 μg/ml) on MSCs subjected to hypoxia for 72 h. A. Morphological changes of MSCs under different treatments; B. LDH release; C. MSC survival assayed as MTs test; D. Ratio of LDH to MTs.

To evaluate the cytoprotective effect of clusterin, MSCs were treated with exogenous clusterin (0–4 μg/ml) when MSCs were exposed to hypoxia. Administration of clusterin significantly reduced the numbers of dying and dead cells (Fig 4A). Moreover, LDH release was reduced and MTs uptake was increased in a concentration dependent manner (Fig 4B–4D).

### Clusterin maintained mitochondrial function

**Fig 5:**
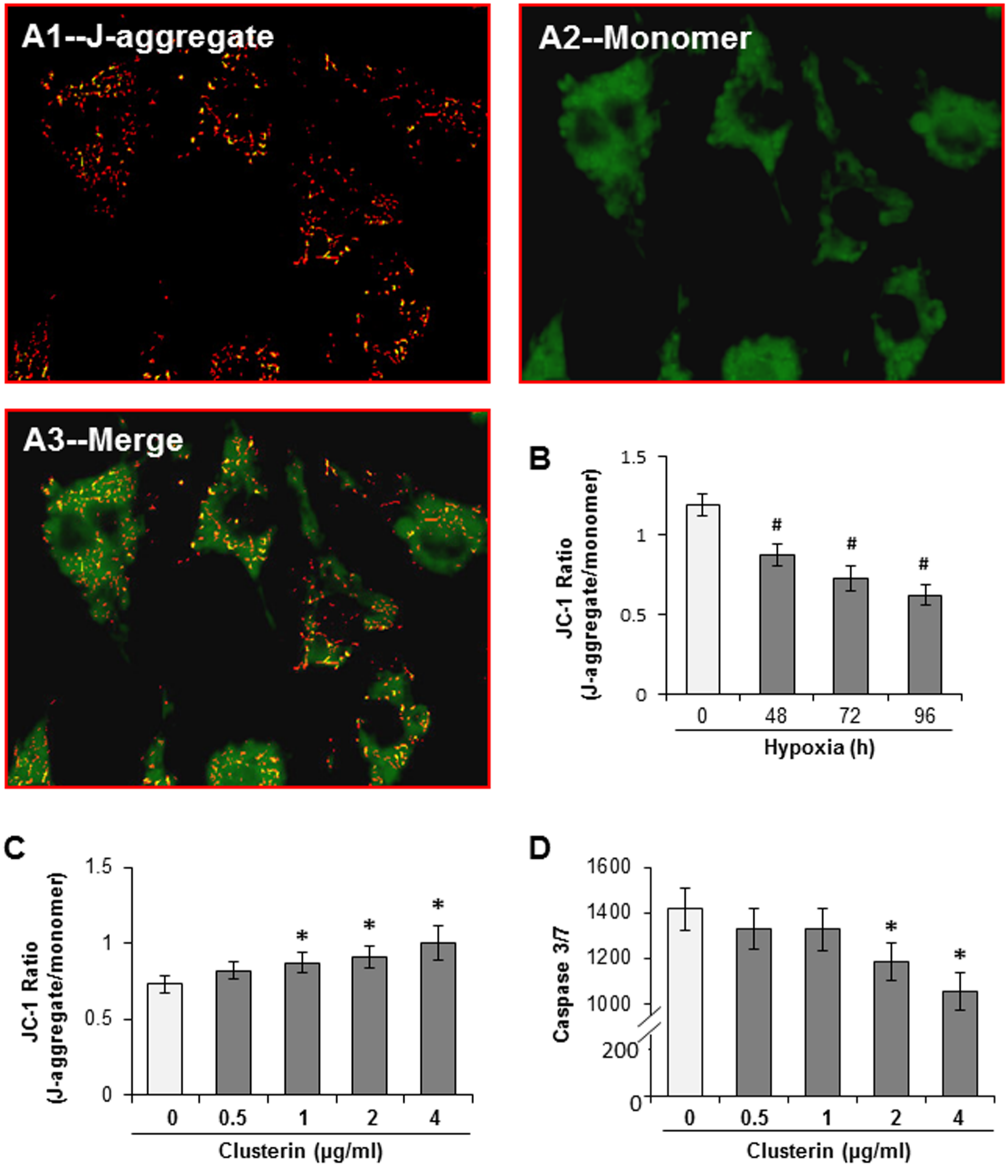
ΔΨm and activity of caspase3/7 in MSCs exposed to different environments. A. JC-1 fluorescence imaging in MSCs. The green fluorescence represented the JC-1 monomer and red fluorescence represented the JC-1 aggregate image. B. JC-1 ratio of J-aggregate to monomer in MCSs exposed to hypoxia for different time. C. Effect of clusterin on JC-1 ratio of MSCs cultured under hypoxia for 72 h. D. Effect of clusterin on the activity of caspase 3/7 in MSCs cultured under hypoxia for 72 h. ^#^, *p* < 0.05 *vs* normal control; *, *p* < 0.05 *vs* hypoxic control without clusterin, respectively.

When JC-1 was loaded into MSCs, cells exhibited a characteristic distribution pattern of hypo- (green fluorescence of monomer) and hyper- (red fluorescence of J-aggregate) polarized mitochondria (Fig 5A). The red fluorescent mitochondria were confined to the cell periphery, whereas the green fluorescent mitochondria were localized near the nucleus. The ratio of JC-1 J-aggregate to monomer fluorescence was 1.146 ± 0.081 in MSCs cultured in normal condition. After cells were subjected to hypoxia, the distribution pattern of hypo- and hyper-polarized mitochondria didn’t exhibit changes. However, the JC-1 ratio was reduced in a time dependent manner (ΔΨm decreased) (Fig 5B). The JC-1 ratio was well maintained in MSCs treated with exogenous clusterin during exposure to hypoxia for 72 h compared to that in cells cultured alone (Fig 5C). The activity of Caspase 3/7 was also significantly reduced in MSCs treated with clusterin (> 2 μg/ml) (Fig 5D).

### Signaling pathway of clusterin mediated cytoprotection

**Fig 6:**
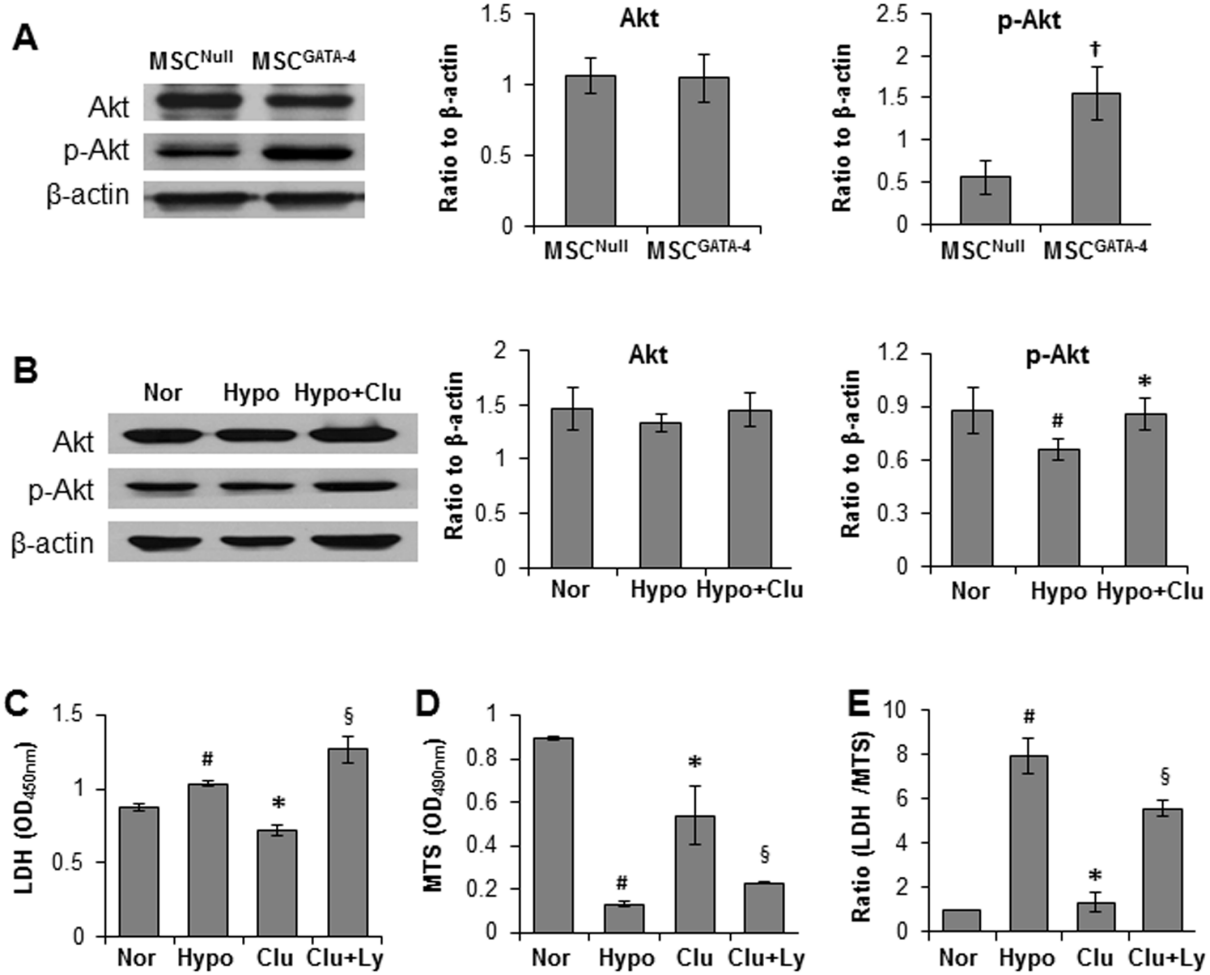
Relationship of clusterin with Akt triggered cytoprotection against hypoxia. A & B. The representative western blots and semi-quantitative estimation of total Akt and p-Akt in MSCs with different treatments. C-E. PI3K inhibitor LY294002 (5μM) abrogated the cytoprotective effect of clusterin on MSC against exposure to hypoxia for 72 h. ^†^, p<0.05 *vs* MSC^Null^; ^#^, *p* < 0.05 *vs* normoxia control; *, *p* < 0.05 *vs* hypoxia control; ^§^, *p* < 0.05 *vs* hypoxia + Clu. Clu = clusterin; Clu + Ly = clusterin + LY294002.

Akt activity was determined in MSCs exposed to different treatments. No significant differences in total Akt was detected among MSC^Null^, MSC^GATA-4^ and MSCs cultured under normoxia, hypoxia and hypoxia + clusterin (2 μg/ml) (Fig 6A and 6B). The expression of p-Akt was significantly increased in MSC^GATA-4^ compared to that in MSC^Null^. However, the expression of p-Akt was reduced in MSCs exposed to hypoxia for 72 h and well preserved in clusterin treated cells. Moreover, the PI3K inhibitor LY294002 (5μM) significantly diminished clusterin (2μg/ml) mediated reduced LDH release and increased MTs uptake (Fig 6C–6E).

## Discussion

Our previous studies have indicated that overexpression of GATA-4 successfully increases MSCs transdifferentiation [21] and survival in ischemic myocardium [9]. In the present study, we demonstrate using a cell culture model system that clusterin is partially responsible for increased cell tolerance of MSC^GATA-4^ to ischemia. The cytoprotective effect of clusterin may be associated with the maintenance of mitochondrial membrane stability through activation of the Akt signaling pathway.

### 1. Upregulation of clusterin in MSCs is involved in GATA-4 mediated cytoprotection

Our study indicates that overexpression of GATA-4 significantly increases MSC survival in ischemic environment, which is consistent with our previous report [9]. The expression of clusterin was significantly upregulated in MSC^GATA-4^ compared to that in MSC^Null^. It is well known that clusterin is widely distributed in many tissues and organs, where it participates in different biological processes. To ascertain the role of clusterin in cell survival, cells were transfected with Clu-siRNA. The survival of MSC transfected with Clu-siRNA was significantly lower compared to that of negative control si-RNA transfected cells. This has been substantiated by direct supplementation of exogenous clusterin to MSC and its effect on MSC survival. Clusterin is a multifunctional glycoprotein and protects cells against both apoptosis and necrosis induced by genotoxic stress and oxidative stress [12]. It has been previously reported that the cell death of retinoblastoma cells due to cisplatin was prevented by 5 μg/ml of clusterin [15]. Silencing clusterin gene expression in OS cells by small interfering RNA induces spontaneous apoptosis and reduced growth ability [22]. It has been reported that clusterin secretion from Sertoli cells protects the testes from heat stress-induced injury [13]. Clusterin expression in oxygen-glucose deprivation conditions may play a protective role against ischemia-induced loss of tight junction protein and human retinal endothelial cells death [14]. Intravenous clusterin administration after myocardial infarction in rat has been demonstrated to reduce infarct size and improved survival [18].

### 2. Clusterin maintains mitochondrial function through activating Akt signaling pathway

The mechanism of clusterin mediated cytoprotection is not yet clear. It has been suggested that the effect of clusterin on myocardial infarction is independent of clustein binding with its receptor, megalin [18]. It has been reported that the cytoprotective effect of clusterin was associated with cell cycle progression and DNA damage repair [23]. Our results show that MSCs exhibit a characteristic distribution pattern of hypo- and hyper- polarized mitochondria. After cells were subjected to hypoxia, the ratio of hyper- to hypo- polarized mitochondria was reduced in a time dependent manner (ΔΨm decreased). However, ΔΨm was well maintained in MSCs treated with exogenous clusterin during exposure to hypoxia for 72 h and the activity of Caspase 3/7 was also significantly reduced. These results are consistent with the notion that clusterin protects cells against apoptosis by inhibiting the dissipation of mitochondrial potential and preventing the release of cytochrome-c from mitochondria into the cytoplasm as well as the subsequent activation of the caspase cascade [24]. Mitochondria generate most of the cell's supply of ATP by utilizing the proton electrochemical gradient potential (Δp). The total force driving protons into the mitochondria (i.e., Δp) is a combination of both ΔΨm and the mitochondrial pH gradient [25]. During cellular stress, ΔΨm may in turn be altered, resulting in mitochondrial dysfunction, cellular metabolism interruption, and initiation of apoptosis. Therefore, our data is in agreement with that clusterin maintains mitochondrial membrane potential and stabilizes membrane permeability.

The protein kinase Akt is identified as an important survival factor that suppresses the mitochondrial pathway of apoptosis [26]. It has been reported that clusterin contributes to increased cell resistance by triggering the activation of Akt and GSK-3β [19, 27]. In the present study, we observed that clusterin-mediated cytoprotection is associated with increased phosphorylation of Akt. Our study indicated that Akt was activated via a PI3-K-dependent pathway because the inhibitor of this pathway, LY-294002, abolished clusterin-mediated Akt activation. Moreover, the protective effect of clusterin on cell injury is abrogated by PI3K inhibitor LY294002; which may effectively suppress clusterin-induced activation of Akt. Activated Akt promoted the phosphorylation of Bad and resulted in a decrease of cytochrome c release from mitochondria. Collectively, these results suggest that clusterin plays a role in protecting MSCs from oxidative stress and that Akt signaling mediates cell protection by clusterin [19].

## Conclusions

We observed a significant increase of clusterin in MSCs following GATA-4 transfection. Clu-siRNA partially abrogated MSC^GATA-4^ tolerance to hypoxia stress. In contrast, MSCs treated with exogenous clusterin significantly reduced cell injury following hypoxia. These results suggest that clusterin upregulated in MSC^GATA-4^ is partially responsible for GATA-4 mediated cytoprotection. The results of this study suggest that clusterin may be therapeutically exploited in stem cell based therapy for cardiovascular disease.
